# Self-Reported Health Experiences of Children Living with Congenital Heart Defects: Including Patient-Reported Outcomes in a National Cohort Study

**DOI:** 10.1371/journal.pone.0159326

**Published:** 2016-08-03

**Authors:** Rachel Louise Knowles, Valerija Tadic, Ailbhe Hogan, Catherine Bull, Jugnoo Sangeeta Rahi, Carol Dezateux, UK Collaborative Study of Congenital Heart Defects (UKCSCHD)

**Affiliations:** 1 Life Course Epidemiology and Biostatistics Section, Population Policy and Practice Programme, UCL Institute of Child Health, University College London, London, United Kingdom; 2 Cardiac Unit, Great Ormond Street Hospital for Children NHS Trust, London, United Kingdom; University of Barcelona, SPAIN

## Abstract

**Background:**

Understanding children’s views about living with congenital heart defects (CHDs) is fundamental to supporting their successful participation in daily life, school and peer relationships. As an adjunct to a health and quality of life outcomes questionnaire, we asked school-age children who survived infant heart procedures to describe their experiences of living with CHDs.

**Methods:**

In a UK-wide cohort study, children aged 10 to 14 years with CHDs self-completed postal questionnaires that included an open question about having a ‘heart problem’. We compared the characteristics of children with more and less severe cardiac diagnoses and, through collaborative inductive content analysis, investigated the subjective experiences and coping strategies described by children in both clinical severity groups.

**Results:**

Text and/or drawings were returned by 436 children (246 boys [56%], mean age 12.1 years [SD 1.0; range 10–14]); 313 had less severe (LS) and 123 more severe (MS) cardiac diagnoses. At the most recent hospital visit, a higher proportion of the MS group were underweight (more than two standard deviations below the mean for age) or cyanosed (underweight: MS 20.0%, LS 9.9%; cyanosed: MS 26.2%, LS 3.5%). Children in the MS group described concerns about social isolation and feeling ‘different’, whereas children with less severe diagnoses often characterised their CHD as ‘not a big thing’. Some coping strategies were common to both severity groups, including managing health information to avoid social exclusion, however only children in the LS group considered their CHD ‘in the past’ or experienced a sense of survivorship.

**Conclusions:**

Children’s reported experiences were not dependent on their cardiac diagnosis, although there were clear qualitative differences by clinical severity group. Children’s concerns emphasised social participation and our findings imply a need to shift the clinical focus from monitoring cardiac function to optimising participation. We highlight the potential for informing and evaluating clinical practice and service provision through seeking patient-reported outcomes in paediatric care.

## Introduction

The majority of children born today with congenital heart defects (CHDs) survive into adulthood, are able to participate in family and social life and can access education without additional support. Nevertheless living long-term with a surgically corrected cardiac defect can have a significant impact on children’s health and quality of life (QoL)[1,2] and is associated with stress for the individual child and wider family.[3] Lazarus and Folkman[4] proposed that the stress response includes cognitive appraisal of the stressor, therefore the experience of stress and subsequent adaptation in response to chronic conditions varies with the type or stage of illness[5], as well as the child’s stages of development. Moreover the coping strategies that individuals develop through childhood in response to the stress of living with a CHD can contribute to observed differences in their self-reported QoL outcomes.[1,6,7] It is therefore essential that children are supported by health professionals and carers to develop positive responses that optimise their long-term health and wellbeing. Capturing the child’s own perspective on the impact of their CHD on daily activities, rather than those of their parents, teachers or doctors, is fundamental to understanding and promoting positive adaptation.[8]

We used the opportunity provided by a UK-wide multi-centre prospective cohort study, investigating health and QoL outcomes of children born with CHDs, to ask children aged 10–14 years to self-report on their health experience.[9,10] A postal survey was sent to survivors, including separate questionnaires for the child and their parents, thus providing a cross-sectional ‘snapshot’ of children’s health outcomes towards the end of primary (junior) school or start of senior school. All children attended a cardiologist at least once every two years and most attended normal school without additional educational support. This is a time when CHD disease progression is usually stable and children are entering adolescence, however it is also a period of rapid psychological, emotional and social change, characterised by the increasing importance of friends, growing independence and taking greater responsibility for self-care.[11–13]

Our aim was to explore children’s self-reported experiences of coping with CHDs with a view to informing the types of support that could be offered by clinicians and carers to facilitate positive coping strategies. Previous studies have often focused on the experiences of children with moderate and severe CHDs attending hospital clinics[14–16]. In contrast we wished to include the perspectives of children with mild or corrected CHDs who may experience few physical restrictions or educational challenges in their daily lives. In this paper we present the results of a collaborative inductive content analysis of the text narratives and drawings submitted by children with CHDs participating in our cohort study. We defined two ‘clinical severity’ groups using objective clinical measures, such as underlying cardiac diagnosis, physiological functioning of the heart and current clinical management, and compared the self-reported experiences of children within these clinically-defined groups. We hypothesised that children in the ‘less severe’ group would view themselves as ‘healthy’ and those in the ‘more severe’ group as ‘sick’ and that each group would describe different experiences of daily living and demonstrate adaptive responses that reflected these contrasting identities.

## Materials and Methods

The cohort study was established as a study of prenatal diagnosis for CHD[17] and involved 17 paediatric cardiac centres in the UK-wide follow-up of almost 4000 children, born 1992 to 1995, to monitor survival and QoL with serious CHDs.[9] Each child within this cohort has been assigned a primary cardiac diagnosis using a hierarchical classification (adapted from Wren[18]), and this cardiac diagnosis, along with information about clinical management, was used to assign children to a ‘clinical severity’ group (Table 1). The ‘less severe’ group was characterised by children who had experienced surgical repair of their CHD and required no ongoing medical or surgical treatment other than infrequent hospital follow-up. Children with more complex and severe CHDs, that can be surgically palliated but not repaired, will require more frequent contact with health services, often take regular medication and have poorer cardiac function resulting in exercise limitation; we defined this group as ‘more severe’.

**Table 1:** Defining Severity Groups.

**LS: Less Severe**
Children whose surgical or catheter procedures were presumed to have corrected their cardiac defect ***and*** who were not on regular medication. These children had Cardiac Prognostic Severity (CPS) scores of 0 or 1. These children have a cardiac defect that can be corrected by surgery during infancy and this will leave them with a midline chest scar in almost all cases. Subsequently they are likely to have infrequent contact with health services and are unlikely to experience significant physical limitations or educational challenges.
**MS: More Severe**
Children whose surgery was palliative or staged (CPS score 2) ***or*** who required regular cardiac medication. Children with severe and complex cardiac defects may never achieve normal physical health, although infant cardiac surgery may palliate their problem. These children will require further interventions after infancy, regular medication and regular hospital visits (‘more severe’ group). Some children with severe CHDs will continue to have low blood oxygen (cyanosis), which gives the lips and extremities a blue appearance, and they will have a chest scar.

From 2004 to 2007, we received completed questionnaires from 515 (15%) of 2963 surviving children, then aged 10–14 years, who were contacted via their local cardiologists; clinical information was also obtained from individual case-notes review performed by local clinicians.[19] The characteristics of children who were successfully contacted and returned a questionnaire were similar to non-participating cohort survivors (n = 2527; Table 2). Parents (including carers acting in a parental role) completed a questionnaire about their own employment and education, and their child’s health, schooling and daily activities. Children completed a postal questionnaire[10], which included the standardised PedsQL[20] instrument, as well as the open question, ‘What is it like being a child with a heart problem?’, which provided an opportunity for children to add free text. The unstructured format allowed children to report additional experiences or highlight the issues that they considered important but were not captured elsewhere. Recognising that literacy problems and specific learning difficulties occur more frequently in children with CHD, for example as a complication of cardiac bypass during surgery or because the CHD is part of a syndrome associated with developmental delay, children were also invited to draw rather than write a response if they preferred.

**Table 2:** Characteristics of children who did and did not submit narrative text or drawings.

	Children with CHDs who submitted narratives		UKCSCHD cohort survivors who did not submit narratives	
	N = 436		N = 2527	
	N (%)	Missing [N (%)]	N (%)	Missing [N (%)]
**Sex**		0		55 *(2%)*
Male	246 *(56*.*4%)*		1403 *(55%)*	
**Ethnic group**		4 *(<1%)*	Not recorded	
White	420 *(96%)*			
Not white	12 *(2%)*			
**Year of birth**		0		0
1992	31 *(7*.*1%)*		257 *(10*.*2%)*	
1993	153 *(35*.*1%)*		829 *(32*.*8%)*	
1994	126 *(28*.*9%)*		809 *(32*.*0%)*	
1995	126 *(28*.*9%)*		632 *(25*.*0%)*	
**Cardiac Prognostic Severity (CPS)†**		0		9(<1%)
Curative	93 *(21*.*3%)*		676 *(26*.*8%)*	
Corrective	248 *(57*.*1%)*		1426 *(56*.*4%)*	
Palliative	95 *(21*.*8%)*		416 (16.5%)*	
**Severity group**		0	Not estimated^§^	
LS (less severe)	313 *(71*.*8%)*			
MS (more severe)	123 *(28*.*2%)*			
**Primary cardiac diagnosis**^‡^		0		0
Hypoplastic left heart /mitral atresia	7 *(1*.*6%)*		42 *(1*.*7%)*	
Tricuspid atresia	7 *(1*.*6%)*		34 *(1*.*3%)*	
Double inlet ventricle	11 *(2*.*5%)*		42 *(1*.*7%)*	
Pulmonary atresia + intact ventricular septum	13 *(3*.*0%)*		44 *(1*.*7%)*	
Pulmonary atresia + ventricular septal defect	19 *(4*.*4%)*		76 *(3*.*0%)*	
Truncus arteriosus	14 *(3*.*2%)*		39 *(1*.*5%)*	
Complete atrioventricular septal defect	18 *(4*.*1%)*		275 *(10*.*9%)*	
Transposition of the great arteries	99 *(22*.*7%)*		399 *(15*.*8%)*	
Tetralogy of Fallot	37 *(8*.*5%)*		254 *(10*.*1%)*	
Total anomalous pulmonary venous connection	17 *(3*.*9%)*		97 *(3*.*8%)*	
Ventricular septal defect	80 *(18*.*3%)*		588 *(23*.*3%)*	
Aortic stenosis	19 *(4*.*4%)*		66 *(2*.*6%)*	
Pulmonary stenosis	32*(7*.*3%)*		143*(5*.*7%)*	
Coarctation of the aorta	49*(11*.*2%)*		309*(12*.*2%)*	
Miscellaneous^#^	14*(3*.*2%)*		119*(4*.*7%)*	

* includes 5 transplants

^#^ includes defects found in fewer than 40 children in the cohort: congenitally corrected transposition of the great arteries (n = 24), partial atrioventricular septal defect (n = 20), aortopulmonary window (n = 26), atrial septal defect (n = 36) and rarer diagnoses.

‡ Primary cardiac diagnosis[9] was developed from a hierarchical classification adapted from Wren et al.[18]

^**§**^ not estimated for children whose parents did not complete questionnaires as recent information about current regular medication not available

† **Cardiac Prognostic Severity (CPS) score** adapted from Lane *et al* as previously described.[9] **curative**-children who had successful repair of atrial or ventricular septal defect, pulmonary stenosis or total anomalous pulmonary veins and had no additional cardiac defects; **corrective**-children who had a procedure which approximated normal anatomy and restored biventricular function, with no expectation of future surgery during childhood; **palliative**-children whose surgery did not restore biventricular function, including children for whom multi-stage repair was only partially achieved, only a single functional ventricle circulation was possible, who had a valve replacement which required later revision or who had a cardiac transplant.

Descriptive statistics are presented as numbers and percentages; 95% confidence intervals (CI) were estimated using the binomial exact method. Height and weight z-scores were standardised for age and sex against the British 1990 growth reference.[21] These analyses were performed using Stata SE v.11 (StataCorp, College Station, Texas, USA).

### Qualitative analysis

Free text responses were explored using qualitative content analysis[22]; we used an inductive approach involving independent open coding of responses by three researchers, who were masked to the child’s severity group, followed by collaborative revision of these codes until 19 remained. Codes were grouped into categories using NVivo (QSR International Pty Ltd. Version 9, 2010) to reflect the relationships between these and facilitate comparisons. Drawings were coded using a similar approach; most drawings (36/41; 88%) illustrated a written narrative and were assigned a similar code to the accompanying text. Through iterative review, the study team reached a consensus about the themes identified within the data.

The responsible cardiologist and family physician for each child was contacted and informed of the aims and the design of the study. Children gave individual written assent and their parents gave written consent to participation. The study protocol and materials, including the consent and assent forms, procedures and publication of anonymised drawings, were approved by Trent Multi-Centre Research Ethics Committee, UK (Reference: 04/4/017). The study followed the tenets of the Declaration of Helsinki.

## Results

**Fig 1:**
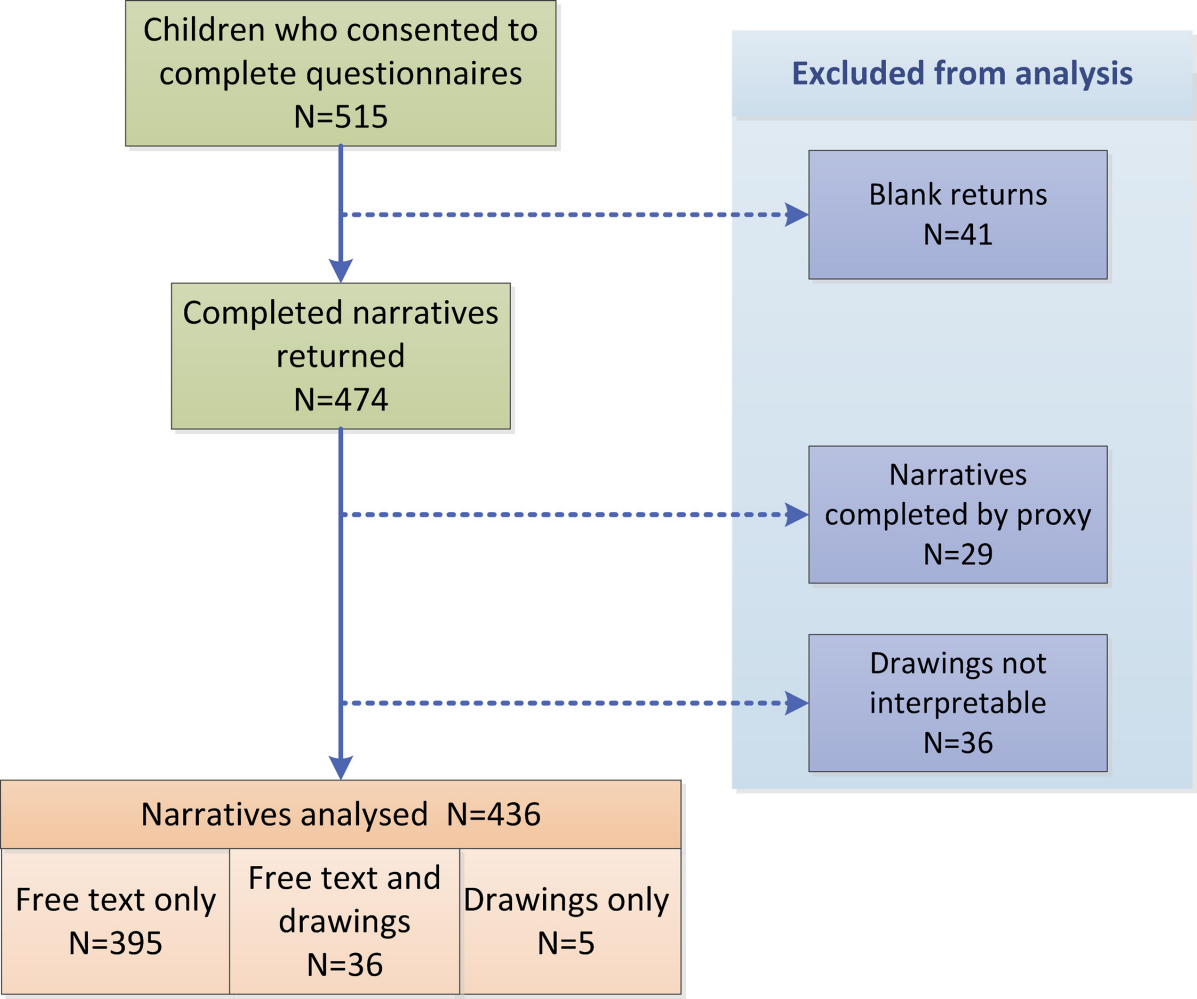
Responses to the questionnaire.

Text and/or drawings were returned by 436 (85%) of 515 children and families who completed questionnaires (Fig 1). Free text responses completed by a proxy (n = 29) and 36 drawings were excluded (one was provided by a proxy, one was bubble-writing text and 34 could not be interpreted). The mean age of children included in the analysis was 12.1 years (SD 1.0 years; range 10–14 years) and 246 (56%) were boys; 313 children (178 boys; 57%) were in the less severe (LS) severity group and 123 (68 boys; 55%) in the more severe (MS) group.

The LS and MS groups did not differ significantly in the proportion of boys or age at completion of the questionnaire (Table 3). Of 123 children in the MS group, 80 (65.0%; 95% confidence intervals [CI] 56.3%, 72.9%) had more than one intra-cardiac defect compared with only 112 (35.8%; 95%CI 30.7%, 41.2%) children in the LS group (difference 29.3% [95%CI 18.9%, 38.6%]). Recent information about height, weight and the presence of cyanosis were available for 207 (47%) of children who attended a hospital clinic when aged between eight and 13 years. The proportion of children who were underweight (defined as more than 2 standard deviations below the mean for age [<-2SD]) was significantly higher in the MS group (LS 9.9%, MS 20.0%; difference 10.1% [95%CI 2.0%, 22.0%]) whereas the proportion with restricted height did not differ significantly between the two severity groups (Table 3). However a significantly higher proportion of children in the MS group were cyanosed compared with the LS group (26.2%, 3.5% respectively; difference 22.6% [95%CI 12.5%, 34.6%]).

**Table 3:** Comparing the characteristics of children within the less severe (LS) and more severe (MS) groups.

	Children in less severe group N = 313			Children in more severe groupN = 123		
Source: Hospital records	N	%	*95%CI*	N	%	*95%CI*
**Sex**						
- Male	178	*56*.*9*	*51*.*3*, *62*.*2*	68	*55*.*3*	*46*.*5*, *63*.*8*
**Associated anomalies**						
- Single cardiac defect	187	*59*.*7*	*54*.*2*, *65*.*0*	37	*30*.*1*	*22*.*7*, *38*.*7*
- Multiple cardiac defects	112	*35*.*8*	*30*.*7*, *41*.*2*	80	*65*.*0*	*56*.*3*, *72*.*9*
- Associated non-cardiac anomalies or syndrome	14	*4*.*5*	*2*.*7*, *7*.*4*	6	*4*.*9*	*2*.*3*, *10*.*2*
**Most recent clinic visit (8–13 years)** *(missing*: *LS = 171*, *MS = 58)*^*§*^						
- Cyanosis	5	*3*.*5*	*1*.*5*, *8*.*0*	17	*26*.*2*	*17*.*0*, *38*.*0*
- Height (<-2SD below mean*)	17	*12*.*0*	*7*.*6*, *18*.*3*	7	*10*.*8*	*5*.*3*, *20*.*6*
- Weight (<-2SD below mean*)	14	*9*.*9*	*6*.*0*, *15*.*9*	13	*20*.*0*	*12*.*1*, *31*.*3*
**Source: Questionnaire**	**Mean (SD)**			**Mean (SD)**		
**Age** *(missing = 0)*						
- At questionnaire completion	12.2 years (SD 1.0)			12.0 years (SD 1.0)		

CI = confidence intervals; SD = standard deviation.

^*§*^Information about height, weight and cyanosis at the most recent clinic visit was available for 207 (47%) of 436 children (142/313 [45.4%] children in Group 1; 65/123 [52.8%] children in Group 2) when aged between 8 and 13 years. (NB Data for other parameters are complete).

*Children with height or weight <-2SD below the mean for age represent fewer than 2.5% of the population and are considered to have poor growth or to be underweight respectively.

**Fig 2:**
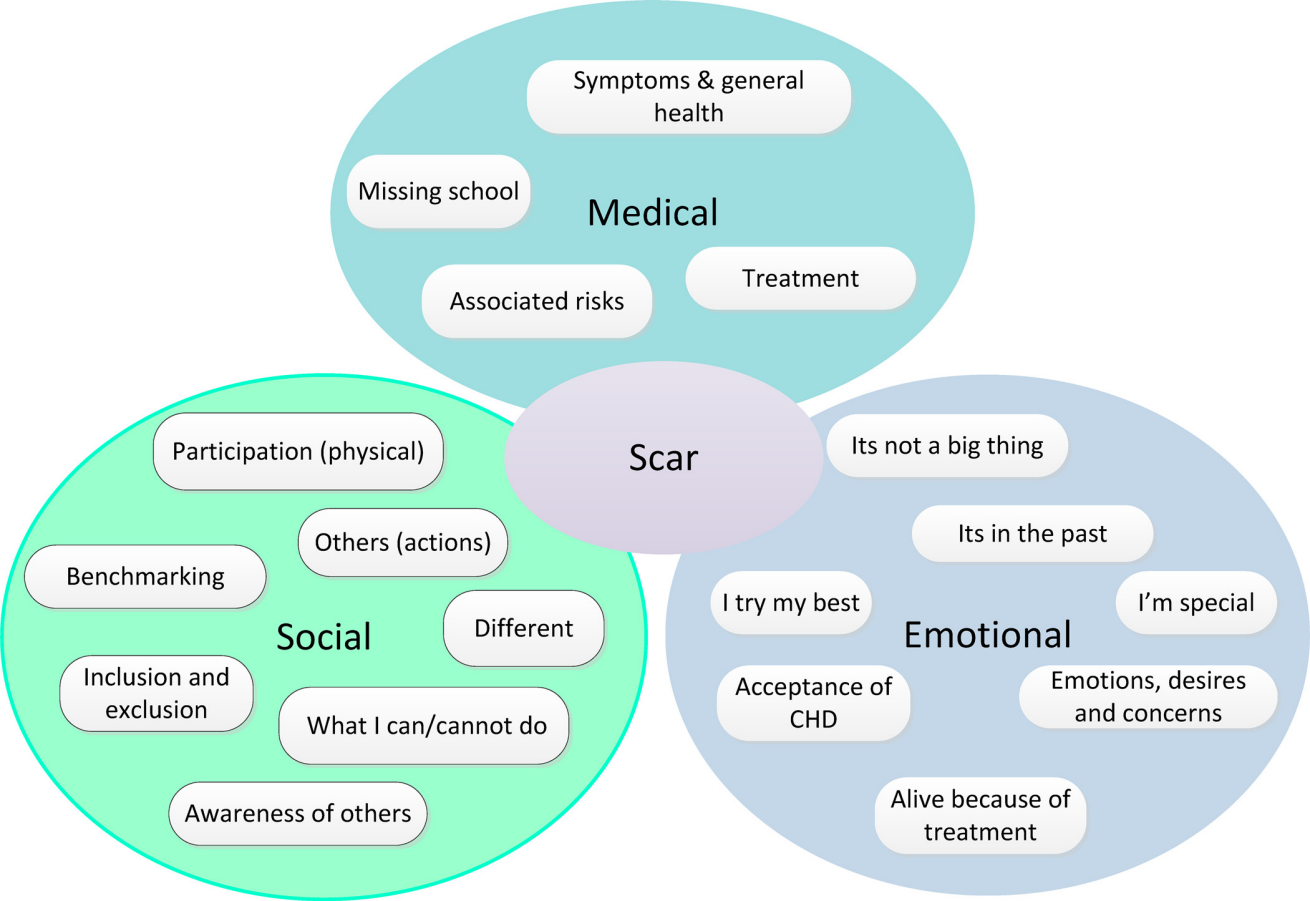
Codes and themes.

Nineteen codes were grouped into three separate categories focused around medical, social and emotional experiences, while the final code ‘Scar’ was associated with all these categories (Fig 2). Overarching themes were developed iteratively and our results are reported under these themes: ‘striving to be the same’, ‘seeking understanding of their illness’, ‘avoiding social exclusion’ and ‘living with risk and uncertainty’. Finally we reviewed the data to identify the different coping strategies developed by respondents and compared the experiences and coping strategies described by children within each severity group. In presenting our results, we describe the themes and coping responses and provide quotes from children’s texts as examples; each quote is followed by the sex and age of the child, and LS (less severe) or MS (more severe) indicating to which clinical severity group they belonged.

### Striving to be the same

Classmates and family members without CHDs were often envisioned as ‘normal’, ‘*everyone treats me like a normal person’* (Boy, 13 years, LS), and used as a point of reference for children when describing the many symptoms that they perceived as related to their heart condition, which included breathlessness, tiredness, chest pains, blue hands or lips, headaches, sore legs, poor appetite and feeling cold: *‘I get colder quicker than my twin’* (Girl, 10 years, LS). Despite choosing different examples to illustrate their experiences, children in both severity groups conveyed a strong aspiration to be like their peers. Children expressed their sense of being different in relation to day-to-day issues such as taking medication, needing more sleep than friends, missing out on school trips and having to be more vigilant about risks to their health. Although they strove to be the same as other children, they recognised that they could never be ‘completely normal’ *‘I don’t know what a normal heart feels like’* (Girl, 12 years, LS).

Often children described their surgical chest scar in terms of the information it provided to others about their heart condition and the risk of being viewed as different. Children frequently did not welcome the attention they received from revealing their scar, ‘*In the changing rooms some children who do not know about my operations are sometimes quite inquisitive which makes me feel a little uncomfortable’* (Boy, 13 years, LS); ‘*[I] get sick of people staring at my scar*, *asking questions all the time’* (Boy, 11 years, MS).

Children in both groups frequently referred to their hospital treatment, surgery and clinic visits. As these played a significant role in framing their identity as patients and therefore different, many expressed strong dislike of the hospital experience, ‘*I hate going to hospital because I hate needles and doctors/nurses*’ (Girl, 12 years, MS), *‘You have to miss school for the doctors*! *Which is very annoying’* (Boy, 12 years, LS). Some children in the MS group seemed more prepared to accept that these hospital visits were part of their lives, especially when further surgery offered the potential to achieve better physical health, *‘I am looking forward to a brighter future after my operation*, *hoping to be fitter*, *run faster and be able*, *hopefully*, *to ride a bike’* (Boy, 12 years, MS).

**Fig 3:**
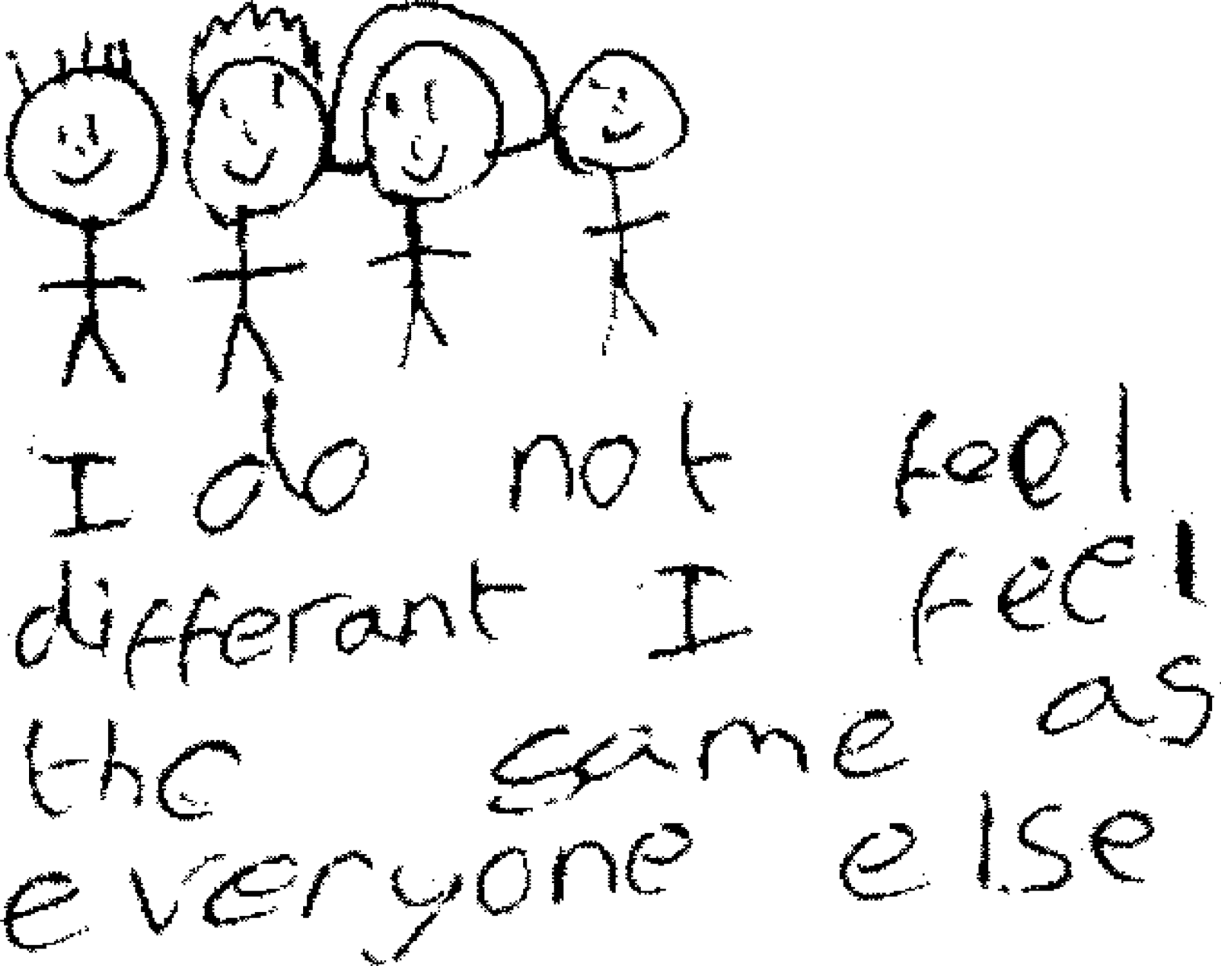
Routine hospital visits.

The routine hospital ‘check-up’ was specifically highlighted by children in the LS group as a tangible reminder that they were different (Fig 3), *‘You’re different from people in the way that you go to hospitals for a regular check-up’* (Girl, 12 years, LS) and taking regular medication was of similar significance in underlining this difference for those in the MS group, *‘[I feel] different from other children*, *because of the medication*…*[at scout camp] Mum always has to fill a medical form in’* (Boy, 11 years, MS).

**Fig 4:**
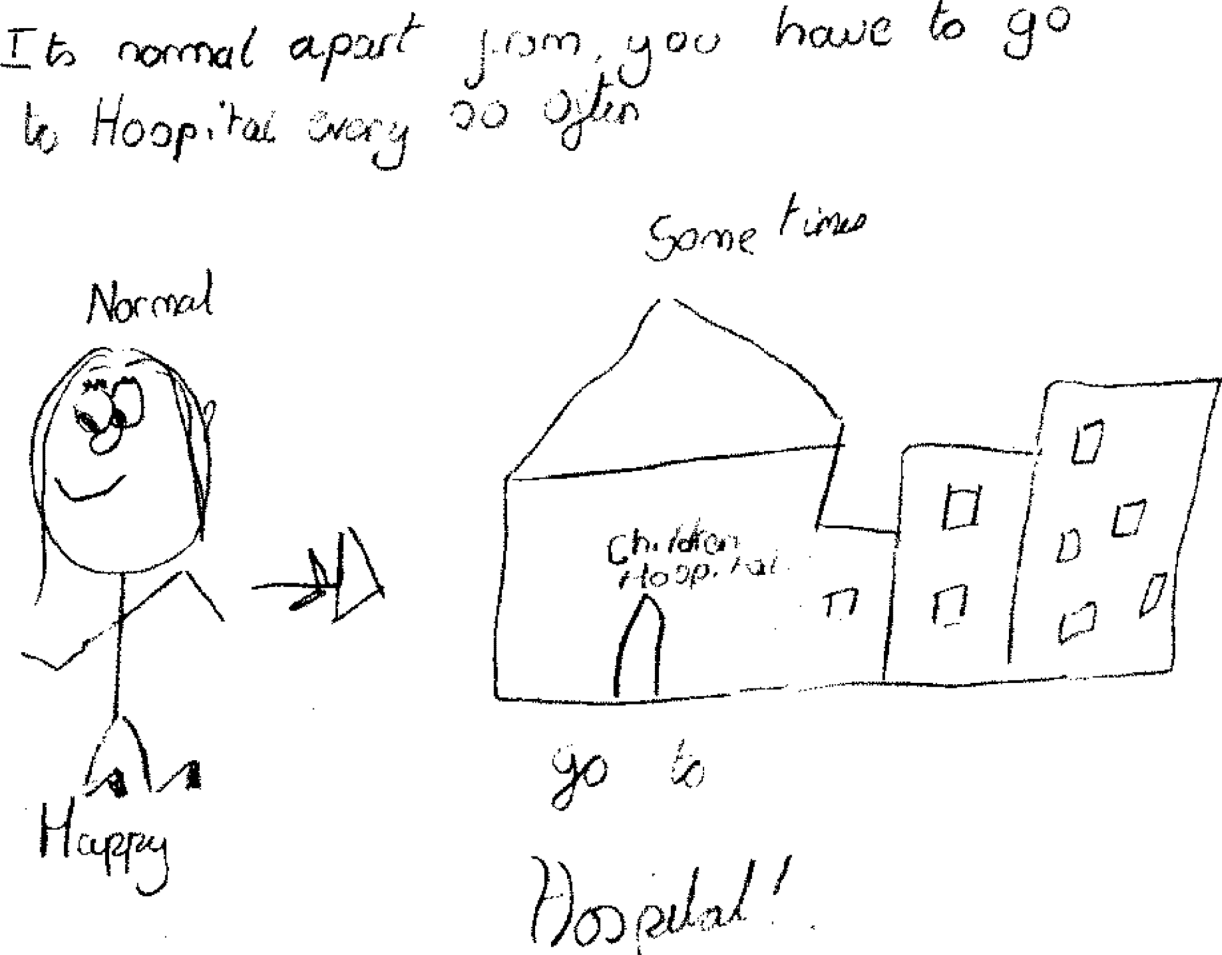
Being the same.

Children’s self-reported narratives often included a strong element of ‘benchmarking’, drawing comparisons between their own abilities and those of other young people who did not have a CHD (Fig 4). Striving to be the same as their ‘normal’ peers was central to their identity, *‘Even though my heart problem restricts me from doing many things I still think of myself as the same as my friends’* (Boy, 12 years, MS) and they regarded themselves as most like other children when they could take part in the same activities, *‘My friends treat me the same as them*, *even though in P*.*E*. *if we are working in groups I can’t always keep up’* (Girl, 11 years, MS). Whenever possible, children within the LS group emphasised activities in which they surpassed their peers either physically or academically, *‘I am usually ahead of the class and finish first at most school work’* (Girl, 11 years, LS); *‘I do a lot of sports like swimming*, *trampolining & kickboxing which are more than my friends can do*’ (Girl, 10 years, LS), whereas only one child in the MS group described doing better than classmates and this was at piano, a less strenuous activity. Although these examples demonstrate that children in both severity groups compared themselves to peers, those in the MS group more frequently referred to themselves as ‘different’ and emphasised things that they ‘could not do’, in contrast to those in the LS group who wrote about being different and the same with similar frequency (Table 4).

**Table 4:** Ranking different codes by frequency of reporting within each clinical severity group.

Less severe	Number of times* code was identified	More severe	Number of times* code was identified
(N = 313 children)		(N = 123 children)	
**Emotional**		**Emotional**	
Emotions, Desires, Concerns	127	Emotions, Desires, Concerns	60
It's not a big thing	111	It's not a big thing	33
Its in the past	29	I try my best	7
Acceptance of CHD	22	Acceptance of CHD	6
I'm special	20	I'm special	5
I try my best	18	Its in the past	3
Alive Because of Treatment	18	Alive Because of Treatment	1
**Medical**		**Medical**	
Treatment	110	Treatment	52
Symptoms/General Health	84	Symptoms/General Health	37
Associated Risks	27	Associated Risks	8
Missing School	9	Missing School	2
**Social**		**Social**	
Participation (physical)	173	Participation (physical)	65
Benchmarking	97	Benchmarking	47
Others (actions)	67	What I can't do	47
Others (awareness)	64	Different	28
What I can do	59	Exclusion\Inclusion	27
What I can't do	56	Others (awareness)	24
Different	41	Others (actions)	22
Exclusion\Inclusion	30	What I can do	10
**Scar**		**Scar**	
Scar	79	Scar	17

*As codes may have been identified more than once within an individual child’s text, percentages have not been calculated.

### Seeking understanding of their illness

When children described the physical symptoms that they experienced, they demonstrated an interest in understanding how these might relate to their CHD. They frequently described breathlessness and were generally confident about attributing this to their cardiac defect even though it was triggered by different types of physical exertion. Children with less severe heart defects described becoming short of breath after significant physical exercise such as running or PE whereas, in contrast, those with more severe defects associated breathlessness with less strenuous everyday activities, for example ‘*singing’* (Girl, 11 years, MS) or *‘carrying a heavy bag’* (Girl, 11 years, MS); this was a marked difference between the two severity groups. Some children within the MS group felt physically unwell even without exertion, *‘You don’t always feel like eating*, *lots of the time you don’t feel great’* (Girl, 12 years, MS).

Children expressed uncertainty about the relationship between some symptoms and their heart condition: ‘*Sometimes my heart does ache’* (Boy, 11 years, LS), ‘*[my asthma] could equally be … why I get breathless and sharp pains in my chest’* (Girl, 11 years, LS), ‘*I suffer from migraines…I think that this is connected?’* (Girl, 13 years, LS). Such statements were more common within the LS group who described fewer symptoms that appeared clearly related to cardiac function.

As well as identifying their CHD as a cause of physical symptoms, children also linked it to educational challenges, such as difficulty understanding instructions, lack of concentration or needing educational support.

### Avoiding social exclusion

**Fig 5:**
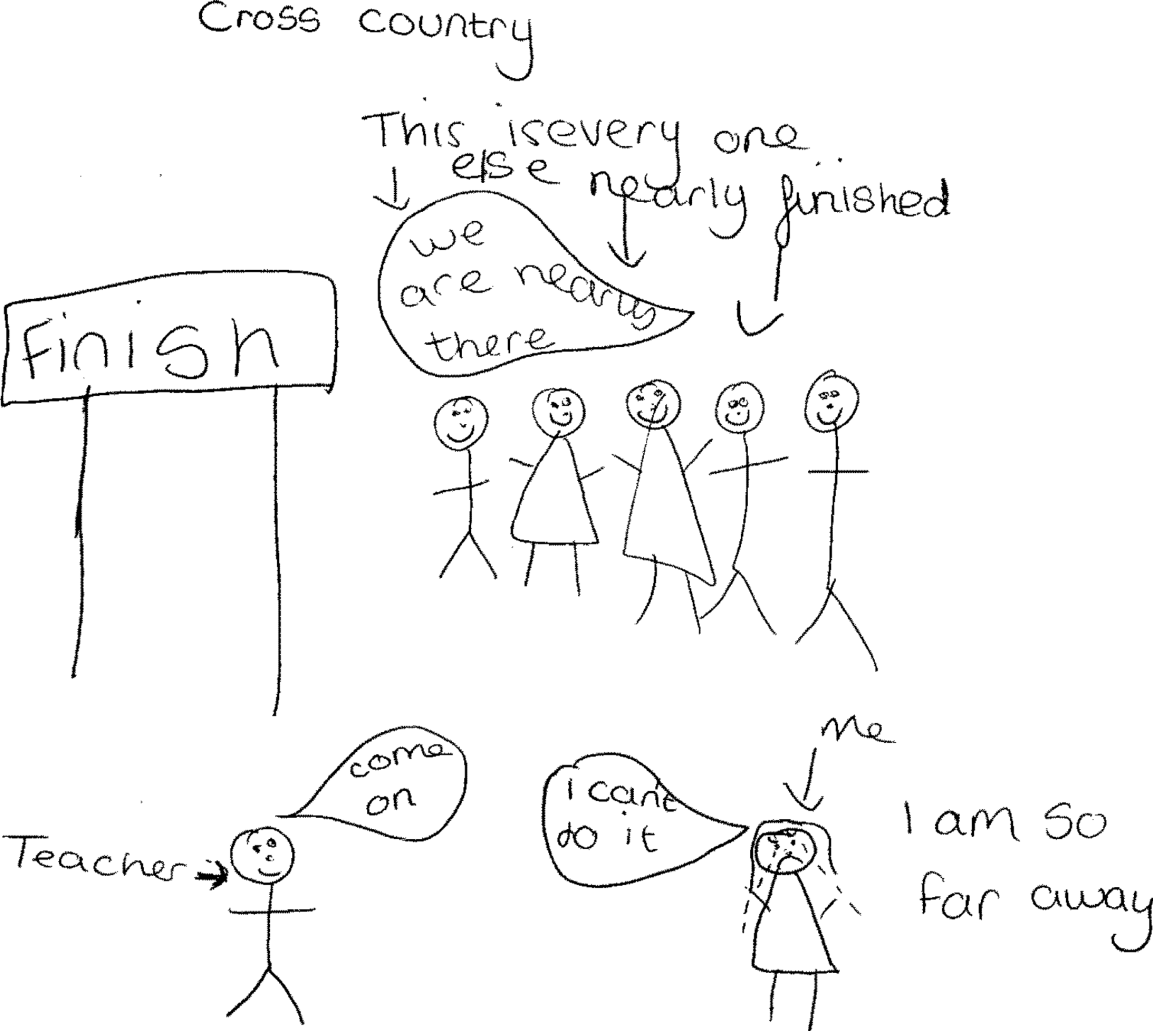
Trying to keep up with friends.

A prominent issue for all children with CHD, regardless of severity, was their sense of belonging to their peer group, which they referred to as ‘fitting in’, ‘joining in’ or–conversely—feeling ‘left out’ or ‘missing out’. The activities that children described in relation to exclusion differed; children in the LS group focused on being involved in sports and adventurous activities, such as rollercoasters or theme parks, while children in the MS group focused on being excluded from school sports or physical education (PE) lessons. Children associated not being able to undertake the same physical activities as their peers with social exclusion (Fig 5), ‘*you cannot play sports and are weaker slower than everyone else…you can get left out a lot’* (Boy, 13 years, MS) and they were anxious not to be excluded, ‘*Some of my classmates even seem to think that I’m completely immobile’* (Boy, 13 years, MS). Being given non-strenuous roles was not regarded as participation, *‘[I] have to help referee…whilst others have fun’* (Girl, 12 years, MS). Children in both severity groups valued the support offered by their peers, and appreciated when friends adjusted their behaviours to ensure they were not excluded due to the physical limitations imposed by their heart condition, *‘My friends [keep me] company on the bench!’* (Girl, 11 years, LS).

Although many children wrote about having good friends, they noted that maintaining these friendships could be difficult, particularly during the transition from primary to secondary school, *‘it’s quite hard to make friends because … you’re different to them… it took almost a year for me to make friends in my high school’* (Girl, 11 years, MS).

Many children expressed feelings of loneliness, *‘[I] feel alone because no one has been through what I’ve been through*’ (Boy, 13 years, LS). They actively managed information about their health to avoid the risk of social exclusion, *‘if I don’t tell them or if they don’t see my scar they don’t even know I had one’* (Boy, 14 years, LS). Children in the LS group resented their inability to participate, *‘I do get very annoyed sometimes because I can’t keep up with my friends’* (Boy, 12 years, LS) whereas, in contrast, those in the MS group described their response to this situation in terms of disappointment and resigned acceptance, *‘I can’t always join in and that makes me sad and unhappy sometimes’* (Boy, 11 years, MS).

**Fig 6:**
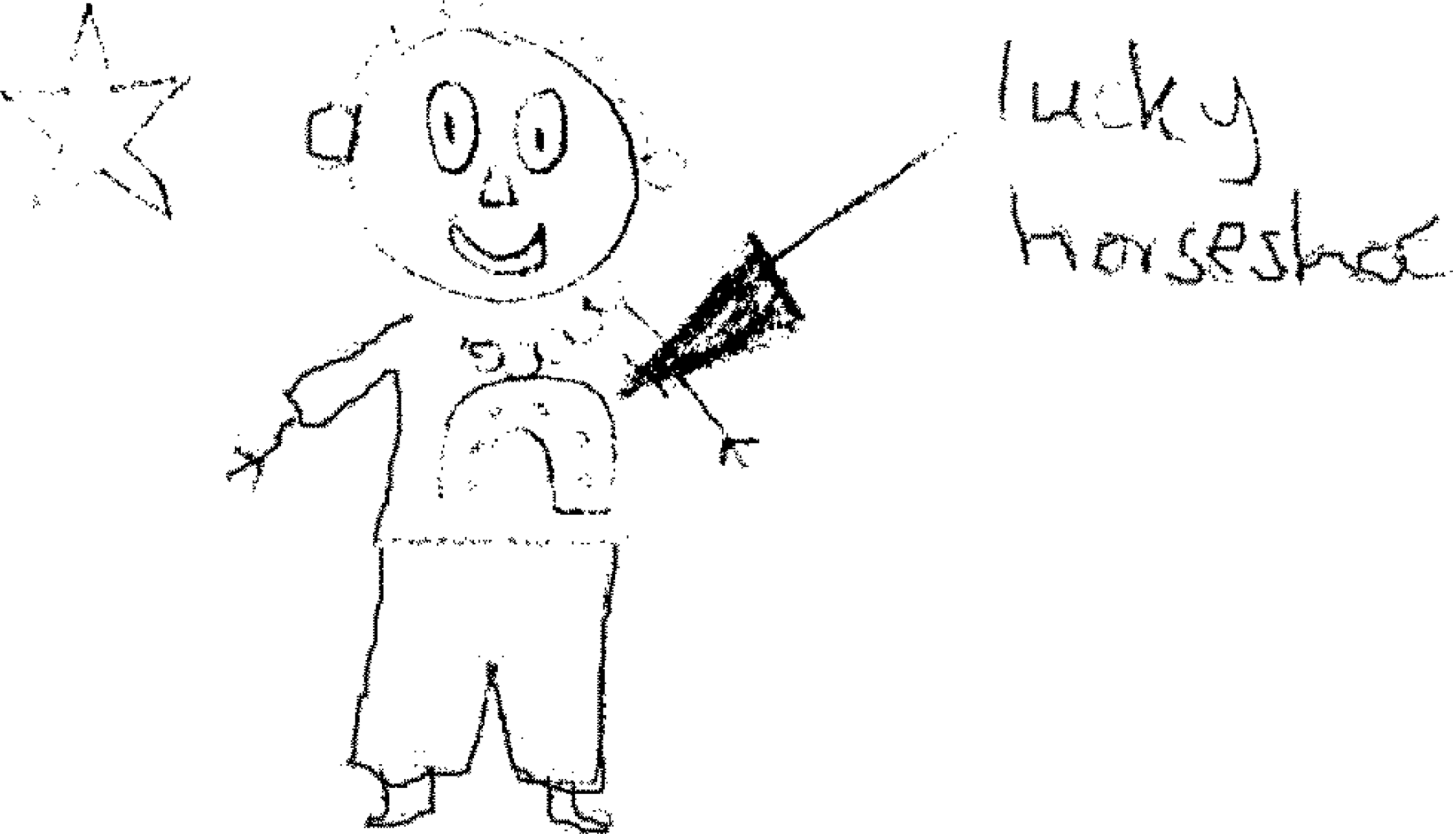
Being a lucky survivor.

Children in both groups demonstrated an acute awareness of other people’s perceptions and described how this could and did affect their social relationships. Thus the scar was highlighted by several children in the LS group as a positive symbol of pride, courage and lucky survival (Fig 6) that made them feel special and promoted social integration, ‘*the scar may impress people giving you lots of friends and also you can make yourself more likely to fit in’* (Boy, 13 years, LS). Only one child in the MS group referred to their scar as associated with ‘luck’. Being ‘special’ was sometimes used to imply a sense of being ‘unique’ but this was often associated with negative characteristics, such as difference and feeling an outsider, *‘I feel different because I’m the only child in school with a big scar on my chest*.’ (Boy, 10 years, LS)

### Living with risk and uncertainty

Children in both severity groups were very aware of potential risks to their health and many described taking precautions to avoid these. They described the serious consequences of infection, particularly in relation to dental treatment or simple cuts, and frequently identified this risk with restrictions on their ability to take part in peer activities, such as having body piercings, ‘*I also would love to get my ears pierced but my heart doctor advised me not to because of maybe getting an infection’* (Girl, 12 years, LS]. Children were cautious about taking part in games in which there might be contact with other children *‘[other children] are careful not to hit me in the chest’* (Girl, 13 years, LS). Theme park rides were highlighted by several children as a risk, *‘I couldn’t go on a [class] trip to Alton Towers…the rides would make my heart beat very fast’* (Girl, 12 years, LS). In the main, these potential health risks were described by children in the LS group as perceived limitations to full participation. Within the MS group children expressed concerns about risks that they were faced with every day. This was exemplified by one child who pointed out the practical importance of informing others about their CHD, ‘*I have to take 6 tablets in one day*. *I have to make sure people know about my heart problem’* (Boy, 10 years, MS) and another, who required warfarin as a regular medication to thin the blood and emphasised the disruptive effect this had on daily activities, ‘*Being put onto Warfarin changes your life because you become scared of getting bumped*, *hurt*, *cut*, *because you bleed a lot or get blood clots’* (Girl, 12 years, MS).

Children in both groups worried about their long-term health, ‘*Sometimes I wish life was really simple because I can get stressed a lot which can sometimes lead to me worrying over nothing…sometimes it gets really tough*. *Sometimes I don’t even know what to do’* (Girl, 13 years, MS). Many focused on uncertainty about their future, particularly in relation to further surgery and physical capacity, *‘‘[I] worry that I’ll have to stop playing [football] when I’m older as it gets more physical*’ (Boy, 10 years, LS) or in new social situations. Hospital appointments could make children who were otherwise active acutely aware of their vulnerability, *‘I am scared … in case they find something wrong with me*. (Girl, 11 years, LS) but it was rare for children to explicitly express fears about dying, *‘It is scary not knowing what will happen to me*. *I am afraid to die young with my heart problem*’ (Boy, 11 years, LS).

As well as health risks, children were concerned about threats to their social participation, thus children in the MS group were concerned about adults limiting their social integration by being over-protective, *‘I get upset with my mum because she treats me like a baby watching over me all the time’* (Boy, 12 years, MS) and a few children described being bullied or teased, *‘[People] call me "Blue Lips" or "Purple Lips"*. *It really upsets me’* (Girl, 12 years, MS). Some children in the LS group developed strategies to avoid being targeted despite having no experience of being bullied, *‘It’s always a good idea to answer questions truthfully*, *and without hesitation or bullies may see it as a weak spot and start teasing’* (Girl, 13 years, LS).

Although children were at a key age for developing concerns about body image[23], this appeared to be a concern for only a few children, and one girl clarified, ‘*[The problem is] not actually with the heart problem*. *It’s with the scar you get left with*. *I would like to wear a strappy top but I don’t because people will see my scar and I think that they will laugh or say how ugly it is*.*’* (Girl, 13 years, MS)

### Coping strategies

**Fig 7:**
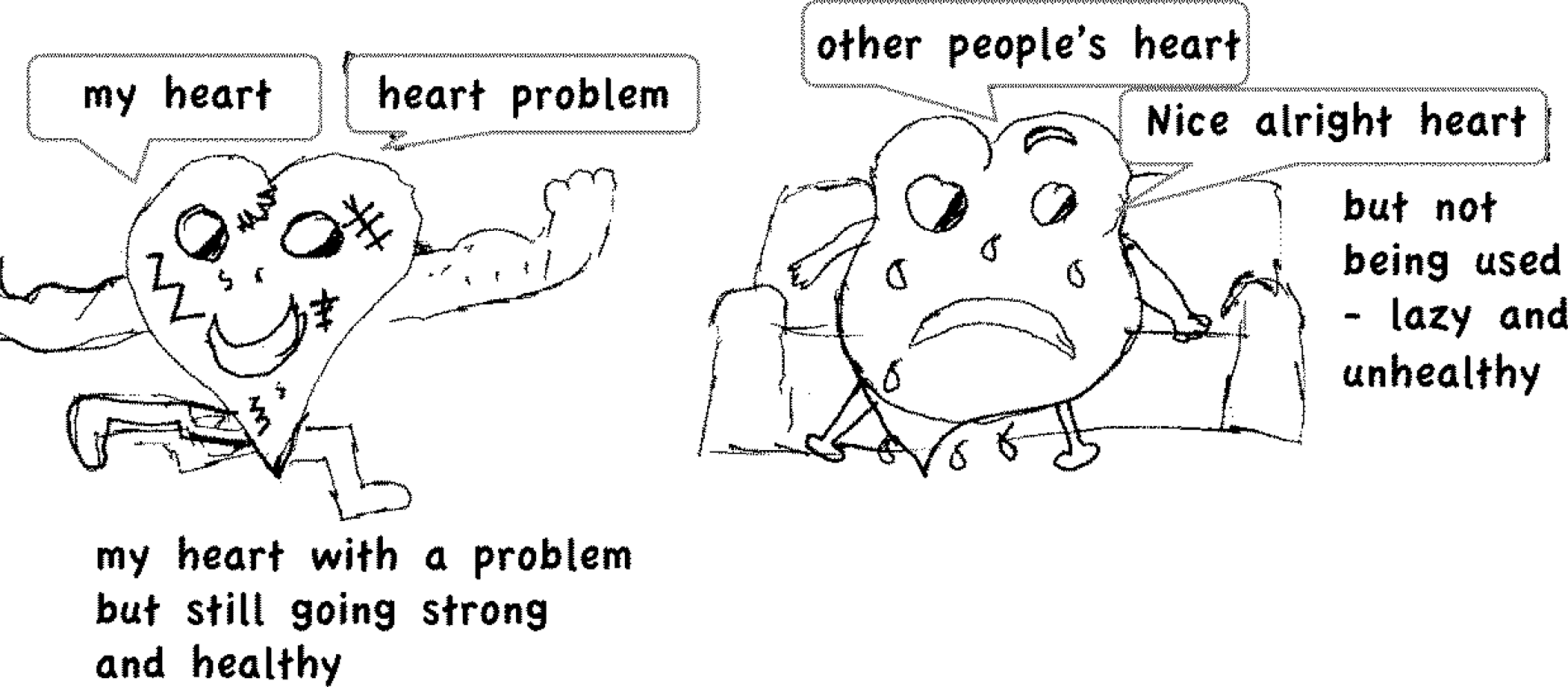
Positive reframing–keeping fitter than others.

As children illustrated the ways in which they tried to make sense of their symptoms and to manage their CHD in the context of their daily lives and social relationships, they also appeared to actively create coherent narratives that fitted with their identity and supported the development of coping strategies to optimise their wellbeing. Despite children living with diagnoses of varying severity, the coping strategies that they developed were often similar for the two severity groups, including accepting their condition as part of their identity (acceptance), emphasising that their experiences are similar to others (normalising) and reassuring themselves that others also find things hard (consolation), and positive reframing (Fig 7), *“the only thing is I can’t go on rollercoaster; but I don’t care*, *who wants to have the time of their life and then puke afterwards*. *Not me*.*”* (Girl, 13 years, LS). Nevertheless some coping strategies, such as the sense of ‘being a survivor’ (Fig 6) or the ability to regard their heart as *“mended”* (Boy, 12 years, LS) so*“what happened to me as a baby…it is in the past”* (Boy, 13 years, LS), were notably absent in children in the MS group. In contrast, only children in the MS group described external sources of support as important to helping them cope with difficulties in their daily lives (Table 5).

**Table 5:** Coping strategies described by children with congenital heart defects.

Coping strategy	Description of coping strategies	Examples
**Coping strategies characteristic of all participants**		
**Positive reframing**	Participants wrote about *‘trying my best’* despite experiencing greater difficulty in physical activities than peers, or described activities at which they excelled. They also described advantages in being able to avoid certain activities.	*‘I can ice skate and play the piano better than they can*.*’ (Boy*, *10 years*, *MS)*.
		*‘you can get out of cross country which is cool*.*’ (Girl*, *11 years*, *LS)*.
		*‘I can’t go on rollercoaster; but I don’t care*, *who wants to have the time of their life and then puke afterwards*. *Not me*.*’ (Girl*, *13 years*, *LS)*.
**Consolation**	Some positive statements were a form of consolation.	*‘I think that I am quite lucky even having a heart problem*. *Because most people die when they are born or have a heart problem when they are still growing*.*’ (Girl*, *12 years*, *MS)*.
**Acceptance**	Some participants coped by accepting their heart condition and adapting their expectations and behaviours.	*‘If I can’t do something then I don’t really bother and I just watch my friends do it’ (Girl*, *12 years*, *LS)*.
		*‘if … ball went off the pitch [my friends] would never make me run and get it*.*’ (Boy*, *12 years*, *MS)*.
	For a few children, their heart problem was not the most difficult problem they had to cope with.	*‘My heart problem is not too much of a problem*, *its my weakness down my right side I find more of a problem’ (Girl*, *11 years*, *MS)*.
**Normalising**	Participants actively attempted to make their experiences normal by presenting them as similar to the experiences of others.	*‘sometimes I get out of breath and tired when I’m running long distances and doing cross country but don’t we all*.*’ (Girl*, *13 years*, *LS)*.
**Seeking knowledge**	Some participants valued the information from routine tests and found additional knowledge reassuring.	*‘I am pleased that I have regular check-ups to make sure everything is still ship-shape*.*’ (Boy*, *13 years*, *LS)*.
**Coping strategies characteristic of all participants (continued)**		
**Managing information**	By hiding or revealing their scar, participants chose to whom they revealed their CHD and exerted some control over how they were perceived by others.	*‘The only way people know I have a heart problem is when I show them my scar*.*’ (Boy*, *13 years*, *LS)*.
	They also expressed concern about ‘losing control’, for example when their diagnosis was revealed by their scar or by others.	*‘Sometimes my friends tell that I have a heart problem and every one crowds round me and sometimes left out and feel very upset and very*, *very sad*.*’ (Girl*, *10 years*, *MS)*.
	Some participants demonstrated effective ways of handling questions to actively define their identity, for example to demonstrate that they were a survivor.	*‘In the school changing rooms when I could see my classmates staring at the scars on my chest and back*, *I gained plenty of attention by regaling them with my frightening tale of a close run in with a ferocious shark on the Great Barrier Reef’ (Girl*, *13 years*, *MS)*.
		*‘When people ask me about my scar*, *I say that … I was lucky to have lived’ (Girl*, *13 years*, *LS)*.
**Coping strategies characteristic of participants in only one of the two severity groups**		
**LS GROUP (Less severe)**		
**Being a survivor**	Some participants showed pride in their ability to survive and referred to themselves as special, unique or lucky. They might attribute their survival to having been ‘rescued’ by doctors and wrote about their gratitude towards clinical staff.	*‘[when] I get asked about my scar*, *I am quite proud and feel special to tell people’ (Girl*, *13 years*, *LS)*.
		*‘the doctors at the hospital were clever enough to fix my heart and to make me feel like I’m a normal child*.*’ (Girl*, *11 years*, *LS)*.
**Distancing**	Some participants regarded their condition as being completed in their past with little impact on their life now.	*‘my parents …told me when I was a baby I had a broken heart but the doctors had mended it*.*’ (Boy*, *12 years*, *LS)*.
		*Because I had my operations when I was only 3 months old I feel normal and believe I fit in at school*. *(Girl*, *12 years*, *LS*.
**MS GROUP (More severe)**		
**External sources of support**	Participants with severe CHD drew on external sources of support, including the special educational needs class and hospice where they felt comfortable. They also valued friends as an important source of support.	*‘My friends help me cope through the times I feel bad*.*’ (Girl*, *13 years*, *MS)*.
		*‘I feel better being in the special class because they’re all like me*. *(Girl*, *11 years*, *MS)*.
		*‘there’s [a] place you can go to like the hospice to get away from reality’ (Girl*, *11 years*, *MS)*.

A central coping strategy for many children regardless of severity group was learning about their cardiac condition and controlling information about it, specifically whether to conceal or reveal its existence to others in order to actively manage the risk of social exclusion. The regular hospital check-up, or situations in which the scar was ‘inadvertently’ revealed were therefore often critical ‘flashpoints’ in young people’s lives, which served as unwelcome reminders of their identity as a patient. Choosing when and with whom to share information about their CHD was a more prominent issue for children in the LS group who appeared able to conceal their CHD, while children in the MS group seemed to accept that others already knew or would find out about this so they described how they might deflect unwelcome questions, for example with humour, *“In the school changing rooms when I could see my classmates staring at the scars on my chest and back*, *I gained plenty of attention by regaling them with my frightening tale of a close run in with a ferocious shark on the Great Barrier Reef!”* (Girl, 13 years, MS).

## Discussion

Children’s self-reported experiences of living with CHDs were provided in response to a large postal survey involving a UK-wide cohort of children with a range of cardiac diagnoses of varying severity. An open question provided an opportunity to capture novel data in an unstructured format from children within a questionnaire that was otherwise comprised of standardised questions about physical health, exercise capacity and QoL. Inductive content analysis provided a suitable methodology for coding and categorising the large volume of data to support thematic analysis and explore the challenges and coping mechanisms faced by children in their daily lives. Notably children’s short written contributions focused on friends/classmates and stressors associated with peer group interactions, with fewer references to their experiences within the family or home. Children strove for social integration by being and doing the same as others, principally their friends; they managed any physical limitations or differences implied by their heart condition to optimise social participation and normality, and to reduce any negative impacts on their daily lives. Although there were many similarities, as hypothesised there were also noticeable qualitative differences in the descriptions of daily life experiences and frequency with which different issues were reported by children within the two clinical severity groups. Thus children in the MS group appeared more willing to accept that they were different, there were things they could not do, and that they had to attend hospital or take medication; children in this group aspired to be included and able to participate alongside peers but were aware of their physical limitations. In contrast children in the LS group referred to their CHD as ‘not a big thing’ and described surpassing peers. Moreover they appeared resentful of specific situations when they were made to feel different, such as not being able to have piercings or having to attend hospital check-ups. In both groups children were actively trying to make sense of their symptoms and to understand their illness, and were concerned to control disclosure of their diagnosis to avoid social exclusion. We identified several coping strategies used by children with CHDs to support social participation, as well as an emphasis on appearing ‘normal’ regardless of the severity of their cardiac diagnosis or any associated physical limitations this implies. Few coping strategies were restricted to only one severity group; notably children in the LS group considered their CHD belonged entirely to ‘the past’ or characterised themselves as ‘lucky survivors’, whereas these adaptive responses were not found in the MS group.

Physical and psychological functioning, subjective experiences and the QoL of children and adolescents with CHDs in comparison with unaffected children, have been previously explored in qualitative and interview-based studies with mixed and sometimes contradictory findings, which do not fully explain which factors predict QoL.[1,2,24] This variability may be related to the developmental stage of child participants[13,25] or methodological differences, for example parent-proxy report has poor correlation with child self-report.[2,26] Uzark noted that cardiac defect severity influenced the psychosocial functioning, but not the physical functioning, component of QoL scales[27] and Silva reported worse QoL for children who had more cardiac operations or residual cardiac functional defects after surgery.[28] Despite this Kendall found no association between severity of cardiac diagnosis and children’s self-perceived health[29], and objective cardiac function or exercise test results do not predict participation in social and physical activities.[30]

Standardised questionnaires are most frequently employed to explore self-reported QoL, health and daily functioning of young people with CHDs, however we were concerned to explore children’s views using a less structured format. Semi-structured interview techniques have been used to elicit subjective experiences of young people with CHDs[14–16], and more recently the focus has been on the transition from paediatric to adult cardiology care in older adolescents aged 16 to 18 years.[12,31–33] As these studies have principally recruited through cardiology clinics and encompass wide age ranges, our study extended previous analyses to capture all levels of clinical severity, including those who rarely attended hospital or experienced few physical limitations, as well as focusing specifically on children in early adolescence. Common findings reported by previous researchers were the importance of social participation[14–16,34,35], an emphasis on achieving normalcy[14], exercise restriction[15,16], awareness about the attitudes of others[15], the significance of managing disclosure[14,15,34], and a relative lack of concern about body image.[15] These views were not dissimilar to those reported by members of our cohort, but we also found that the descriptions from children in the MS group underlined their ongoing struggle to keep up with peers and to be ‘normal’, whereas those in the LS group expressed annoyance on the occasions when they ‘failed’ to achieve as well or be as ‘normal’ as their peers.

Several researchers described children’s adaptive behaviours, such as recognising physical limitations and ‘pacing’, adopting a positive attitude and ‘getting on with it’, humour and self-reassurance.[14–16] Friends were mentioned as an important source of emotional support and companionship[15], however peers were also a potential source of bullying.[16,25,36] Although we identified many of these behaviours in our cohort it was noticeable that some coping strategies, such as distancing themselves from past surgery, were found exclusively in the LS clinical severity group.

Research with children and young people from a range of chronic illness groups has confirmed that patient populations score their QoL lower than do healthy children, and that different QoL domains are impacted depending on the type of disorder.[25,36–38] Children with cardiac conditions tended to score higher than those with cystic fibrosis (CF) and lower than those with diabetes.[38] Research with children affected by CF, which is a life-limiting congenital disorder that impacts on physical activity similar to a CHD, reveals that concerns about social isolation are prevalent and affected children strongly value opportunities and support to participate in physical or social activities.[34,39] In a review of qualitative research, Jamieson et al. found that children with CF gained resilience through taking responsibility for their own health and participating in physical activity[39], and this appeared to be similar for children with CHDs in our study. These findings suggest that supporting independence and promoting shared decision-making for children living with chronic conditions is an important step towards optimising their long-term outcomes. Lightfoot[36] and Kramer[40] have also highlighted the ways in which teachers, parents and classmates can improve school and social environments to enhance inclusion of children with disabilities or chronic conditions.

Controlling information about their diagnosis was an important early step towards taking responsibility for their own health for children with CHDs in our study, and was often associated with other people observing their scar. Interestingly a previous study of children with CHD highlighted concerns about ‘forced disclosure’ of the diagnosis in relation to the inability to take part in physical activity and, as a result, young people became more inactive to avoid the potential risk of stigmatisation.[34] Published research into the outcomes of children living with scars have highlighted that the visibility of a scar, as well as its location and size, influence QoL.[41–44] However the impact of the scar on psychosocial adjustment and QoL appears to be related less strongly to clinically objective measures of severity or size, than to a child’s subjective perception of the significance of the scarring and, where relevant, to psychosocial functioning prior to scarring.[44,45]

Many of our findings were consistent with previous reports although we found little evidence of concerns about future careers[14,15], transition to adult care or body image, but this may have been influenced by the age of children in our study as these experiences have been reported for older adolescents. Our study identified many commonalities but also some significant differences in the experience of children with differing severity of CHDs. Importantly our study provides a valuable snapshot of children’s experiences at the time of transition between primary and secondary school, which is relatively under-researched, yet a time when children are developing essential skills to support their development as an independent social actor and laying the basis for taking responsibility for their own health.

Open questions are often avoided in large surveys because they are complex to analyse, however they have been used successfully in some national surveys to collect respondent perspectives.[46] They also provide an opportunity to elicit novel insights or areas for future enquiry that may not be revealed through a structured questionnaire or interview format, especially in younger age-groups.[47] Our data were extensive and qualitative content analysis allowed us to condense data into individual units of meaning while remaining faithful to the original; crucially, the collaborative and iterative process of developing codes limited researcher subjectivity and reduced potential bias in data interpretation. Nevertheless a limitation of our methodology was that the researcher was not present to explore the concepts underlying an individual child’s responses and there was too little information available to the research team to interpret the meaning of some drawings and narratives. The inclusion of pictures as an acceptable response method was of limited additional benefit as many could not be interpreted independently of accompanying text, however we believe they did have value in encouraging children to respond and in providing children with a means to emphasise and illustrate key issues in their written statements. These limitations must be considered when developing the methodology further.

Nevertheless we highlight that there is considerable added value to including an open question to explore the self-reported patient experiences of children and young people living with CHDs in a large-scale postal questionnaire that is primarily focused on clinical and quantitative outcomes. We have demonstrated that this methodology can contribute valuable information to improve clinicians’ understanding of how young patients with CHD manage their lives and thus inform the provision of clinical services and support. Moreover this approach enabled us to include children with milder disease who had infrequent hospital attendance, and were therefore not well-represented in previous studies recruiting patients at hospital clinics. Traditionally the cardiologist’s focus is on physiology and optimising cardiac function, but it is important to be aware that diagnostic severity only partially influences children’s subjective experiences and adaptation, and care provision should also support educational and social participation. Young cardiac patients, similarly to young people with other types of chronic illness[35] emphasise the challenge of social integration, which is influenced by, but not dependent on, physical performance. Importantly our findings demonstrate the significant and sometimes distressing impact of routine clinical activities, for example hospital visits, on children’s lives. Clinicians should consider how they might best achieve for their patients the optimal balance between essential clinical follow-up and supporting full participation in family and school life.

Our study contributes importantly to the increasing body of research into patient reported outcomes (PROs) in health care and demonstrates the feasibility of embedding PRO measures (PROMs) into a large-scale postal survey. Listening to young patients’ self-reported views is an essential step towards understanding their needs and providing appropriate resources to support them if they are to successfully negotiate the challenges of living with a long-term condition. While an important focus for health research should remain the transition to adult care for young adolescents, our study demonstrates that younger school-age children are also engaged in important social and emotional transitions towards independence as they move towards senior school and develop greater independence from their parents, negotiate new social relationships and continue the journey towards adulthood. In this context, managing information about their diagnosis and deciding with whom they wish to share their cardiac diagnosis appeared to be a pivotal step in assuming greater responsibility for, and sharing in, decisions about their health and self-care. Carers and clinicians should seek the perspectives of children and young people and use these to inform and evaluate clinical support for the development of effective coping behaviours that promote positive health and wellbeing.

## Key Messages

- Children with congenital heart defects (CHDs) often experience physical limitations that impact on daily life activities, education and friendships
- Children’s narratives and drawings illustrate how they make sense of and manage their CHDs and the development of coping strategies to optimise participation in school and social activities
- Managing the disclosure of information about their CHD to other children and adults is an important step for school-aged children in taking responsibility for managing their own health
- Capturing a child’s perspective on the experiences of living with a CHD provides a vital contribution to patient-reported outcomes and understanding patient experiences of healthcare
- Obtaining children’s views through an open-ended question within a postal survey is an innovative technique that could be used more widely to enhance questionnaire-based research, and ensure that the perspectives of young patients help shape service provision and health policy.

## Supporting Information

S1 Table STROBE Statement for Observational Studies. (DOCX)
