# Supplementary material for: Self-Reported Health Experiences of Children Living with Congenital Heart Defects: Including Patient-Reported Outcomes in a National Cohort Study
Source: PLoS One. 2016 Aug 3;11(8):e0159326. doi: 10.1371/journal.pone.0159326 (PMC4972252; doi:10.1371/journal.pone.0159326)
Supplement: S1 Table — (DOCX) [file pone.0159326.s001.docx]

**S1 Table: STROBE Statement for Observational Studies**

|  | |  | Recommendation | Page |
| --- | --- | --- | --- | --- |
| **Title and abstract** | |  |  |  |
|  | | 1 | (*a*) Indicate the study’s design with a commonly used term in the title or the abstract: |  |
|  |  |  | **‘patient-reported outcomes in a national cohort study’** | **Cover page (Title)** |
|  |  |  | **‘self-completed postal questionnaires…collaborative inductive content analysis’** | **Page 2-3 (Abstract)** |
|  |  |  | (*b*) Provide in the abstract an informative and balanced summary of what was done and what was found | **Page 2-3 (Abstract)** |
| Introduction | | | |  |
| Background/rationale | | 2 | Explain the scientific background and rationale for the investigation being reported | **Page 4-5 (Introduction)** |
| Objectives | | 3 | State specific objectives, including any prespecified hypotheses | **Abstract,**  **Page 5 (Introduction)** |
| Methods | | | |  |
| Study design | | 4 | Present key elements of study design early in the paper | **Abstract;**  **Page 5-6 (Patients and Methods)** |
| Setting | | 5 | Describe the setting, locations, and relevant dates, including periods of recruitment, exposure, follow-up, and data collection | **Abstract;**  **Page 5-6 (Patients and Methods)** |
| Participants | | 6 | (*a*) *Cohort study*—Give the eligibility criteria, and the sources and methods of selection of participants. Describe methods of follow-up | **Page 5-6 (Patients and Methods);**  **Figure 1; Table 1** |
|  |  |  | (*b*) *Cohort study*—For matched studies, give matching criteria and number within cohort and comparison group (i.e. classmates) | **Not applicable** |
| Variables | | 7 | Clearly define all outcomes, exposures, predictors, potential confounders, and effect modifiers. Give diagnostic criteria, if applicable | **Page 5-6 (Patients and Methods);**  **Table 1; Table 2** |
| Data sources/ measurement | | 8* | For each variable of interest, give sources of data and details of methods of assessment (measurement). Describe comparability of assessment methods if there is more than one group. | **Page 5-6 (Patients and Methods);**  **Table 1** |
| Bias | | 9 | Describe any efforts to address potential sources of bias | **Page 5-6 (Patients and Methods);**  **Table 1** |
| Study size | | 10 | Explain how the study size was arrived at | **Page 5-6 (Patients and Methods);** |
| Quantitative variables | | 11 | Explain how quantitative variables were handled in the analyses. If applicable, describe which groupings were chosen and why | **Page 5-6 (Patients and Methods); Table 1; Table 2** |
| Statistical methods | | 12 | (*a*) Describe all statistical methods, including those used to control for confounding | **Not applicable** |
|  |  |  | (*b*) Describe any methods used to examine subgroups and interactions | **Not applicable** |
|  |  |  | (*c*) Explain how missing data were addressed | **Not applicable** |
|  |  |  | (*d*) *Cohort study*—If applicable, explain how loss to follow-up was addressed | **Not applicable** |
|  |  |  | (*e*) Describe any sensitivity analyses | **Not applicable** |
| Results | | | |  |
| Participants | 13* | (a) Report numbers of individuals at each stage of study—eg numbers potentially eligible, examined for eligibility, confirmed eligible, included in the study, completing follow-up, and analysed | | **Pages 5-6; Figure 1** |
|  |  | (b) Give reasons for non-participation at each stage | | **Pages 5-6; Figure 1** |
|  |  | (c) Consider use of a flow diagram | | **Figure 1** |
| Descriptive data | 14* | (a) Give characteristics of study participants (eg demographic, clinical, social) and information on exposures and potential confounders | | **Page 6; Table 1** |
|  |  | (b) Indicate number of participants with missing data for each variable of interest | | **Page 6; Table 1** |
|  |  | (c) *Cohort study*—Summarise follow-up time (eg, average and total amount) | | **Page 6; Table 1** |
| Outcome data | 15* | *Cohort study*—Report numbers of outcome events or summary measures over time | | **Not applicable** |
| Main results | 16 | (*a*) Give unadjusted estimates and, if applicable, confounder-adjusted estimates and their precision (eg, 95% confidence interval). Make clear which confounders were adjusted for and why they were included | | **Not applicable** |
|  |  | (*b*) Report category boundaries when continuous variables were categorized | | **Not applicable** |
| Other analyses | 17 | Report other analyses done—eg analyses of subgroups and interactions, and sensitivity analyses | | **Pages 6-13** |
| Discussion | | | |  |
| Key results | 18 | Summarise key results with reference to study objectives | | **Pages 13-14** |
| Limitations | 19 | Discuss limitations of the study, taking into account sources of potential bias or imprecision. Discuss both direction and magnitude of any potential bias | | **Pages 14-15** |
| Interpretation | 20 | Give a cautious overall interpretation of results considering objectives, limitations, multiplicity of analyses, results from similar studies, and other relevant evidence | | **Page 13-15** |
| Generalisability | 21 | Discuss the generalisability (external validity) of the study results | | **Page 15-16** |
| Other information | | | |  |
| Funding | 22 | Give the source of funding and the role of the funders for the present study and, if applicable, for the original study on which the present article is based | | **Page 17** |
